# On Modeling of Plasmon-Induced Enhancement of the Efficiency of Solar Cells Modified by Metallic Nano-Particles

**DOI:** 10.3390/nano9010003

**Published:** 2018-12-20

**Authors:** K. Kluczyk, C. David, J. Jacak, W. Jacak

**Affiliations:** 1 Department of Quantum Technology, Wrocław University of Science and Technology, 50-370 Wrocław, Poland; katarzyna.kluczyk@pwr.edu.pl (K.K.); witold.aleksander.jacak@pwr.edu.pl (W.J.); 2 Madrid Institute for Advanced Studies in Nanoscience, 28049 Madrid, Spain; christin.david@imdea.org

**Keywords:** solar cells, metallic nanoparticles, plasmons, Fermi golden rule, Comsol

## Abstract

We demonstrate that the direct application of numerical packets like Comsol to plasmonic effect in solar cells metallically modified in nano-scale may be strongly inaccurate if quantum corrections are neglected. The near-field coupling of surface plasmons in metallic nanoparticles deposited on the top of a solar cell with band electrons in a semiconductor substrate strongly enhances the damping of plasmons in metallic components, which is not accounted for in standard numerical packets using the Drude type dielectric function for metal (taken from measurements in bulk or in thin layers) as the prerequisite for the numerical e-m field calculus. Inclusion of the proper corrections to plasmon damping causes additional enhancement of the plasmon-induced photo-effect efficiency growth of a metalized photo-diode by ten percent, at least, in comparison to only effect induced by the electric field concentration near metallic nanoparticles. This happens to be consistent with the experimental observations which cannot be explained by only local increases of the electrical field near the curvature of metallic nanoparticles determined by a finite-element solution of the Maxwell–Fresnel boundary problem as given by a numerical system like Comsol. The proper damping rate for plasmons can be identified by application of the Fermi Golden Rule approach to the plasmon-band electron coupling. We demonstrate this effect including the material and size dependence in two types of solar cells, multi-crystalline Si and CIGS (copper-indium-gallium-diselenide) as idealized photo-diode semiconductor substrate modified by various metallic nano-particles, in comparison to the experimental data and Comsol simulation.

## 1. Introduction

Plasmons in metallic nanoparticles have been the focus of growing attention because of their applications in sub-diffraction manipulation of light and related perspectives for the miniaturization of opto-electronic circuits [1,2,3,4,5,6] and advances in photovoltaics [7,8,9,10,11] for the improvement of the efficiency of new generation solar cells via low cost metallic nano-components. The strengthening of the sun-light energy harvesting in solar cells mediated by surface plasmons in metal-nano-particles deposited on photo-active solar cell surface is caused by three effects: (1) the strong concentration of the electric field of the incident photon e-m wave close to the metallic components with a large local curvature (as for small nanoparticles), (2) the large amplitude of plasmon oscillations in metallic nanoparticles (preferring larger nanoparticles with larger number of electrons), and (3) the enhancement of the probability of interband transitions in a semiconductor substrate caused by the breaking of the translational symmetry for a nanoparticle coupled in the near-field regime of surface plasmons with the semiconductor band electrons [1,7,12,13,14,15]. The transition probability for electrons from the valence band to the conduction band in a semiconductor, being essential for the efficiency of the photo-voltaic effect, grows due to the electric field amplitude enhancement and due to the admission of all oblique transitions not prohibited here by the momentum conservation [15]. For the ordinary photo-effect [16], when photons directly induce the interband transitions in a semiconductor, these interband transitions are confined to only vertical ones between states with almost same momentum due to the momentum conservation and the very small momentum of sun-light photons (owing to the large light velocity, $c=3\times 10^{8}$ m/s) which almost does not change the electron momentum at scattering. For the excitation energy ℏω beyond the forbidden gap, $E_{g}$, of the substrate semiconductor, the photon dispersion, ℏω=cq, gives q≪p, where $p∼\frac{\pi \hbar }{l}$ is the semiconductor band quasi-momentum scale in the Brillouin zone (*l* denotes the semiconductor elementary cell linear size). Thus, the change of the band electron momentum $q=p_{2}-p_{1}$ is negligible on the scale of the Brillouin zone and $p_{1}≃p_{2}$ (because of large *c*) and only the vertical, conserving momentum, inter-band transitions contribute to the ordinary photo-effect when the transition is caused by free photons with the momentum q and the energy ℏω=cq (described by the plane-wave, ∼$e^{i(q\cdot r-\hbar \omega t)/\hbar }$).

Nevertheless, in the case of the mediation of the sun-light energy absorption in a semiconductor substrate by plasmons in metallic particles deposited on the surface of a solar cell, the interaction of band electrons with surface plasmons from the metallic nanoparticles deposited on the semiconductor surface changes significantly. In the near-field regime [17,18], the potential of the plasmon dipole in the nanosphere is proportional to $\frac{1}{R^{2}}$ (*R* is a distance from the sphere center), which has a large decomposition in Fourier picture and thus overlaps with all momenta (quasi-momenta) in the substrate semiconductor Brillouin zone. This is in contrary to the potential of the free photon which contributes via only single $e^{i(q\cdot r-\hbar \omega t)/\hbar }$ plane-wave Fourier component in the e-m field vector potential entering the kinematic momentum [16].

The related increase of the light-electron coupling strength via plasmons can be accounted for via the Fermi golden rule (FGR). According to the FGR scheme [19], the probability of the inter-band transitions is proportional to the matrix element of the time-dependent perturbation potential (as the electric field of the photon e-m wave) between an initial and a final state and summed up over all initial states in the valence band and over all final states in the conduction band with the energy conservation imposed, $E_{p}\left(p_{1}\right)+\hbar \omega =E_{n}\left(p_{2}\right)$, where $E_{p(n)}\left(p\right)$ is the valence-*p* (conduction-*n*) band dispersion. The energy ℏω is the excitation energy of surface plasmon oscillations, which have been induced by the incoming photons with e-m wave $∼e^{i\omega t}$ with ω close to the surface plasmon resonance expressed for the metallic nanoparticle as $\omega_{1}=\frac{\omega_{p}}{\sqrt{3}}$ (i.e., the Mie frequency [20,21], $\omega_{p}=\hbar \sqrt{\frac{n_{e}e^{2}}{m^{*}\varepsilon_{0}}}$ is the bulk-plasmon frequency in a metal [22], $n_{e}$ is the density of collective electrons in a metal, $m^{*}$ is the effective mass of electron in a metal, *e* is the electron charge, $\varepsilon_{0}$ is the dielectric constant). The relation between ω and $\omega_{1}$ may be grasped by the damped and forced oscillation scheme displaying the amplitude of plasmon oscillations versus the amplitude of incoming sunlight e-m wave, $D_{0}\left(\omega \right)=const.E_{0}\frac{1}{\sqrt{{\left(\omega_{1}^{2}-\omega^{2}\right)}^{2}+4\omega^{2}/\tau^{2}}}$, where $E_{0}$ is the amplitude of the electric component of the incident photon e-m wave. The amplitude of the surface plasmon dipole, $D_{0}\left(\omega \right)$, is accommodated to the damping of the surface plasmons, $\frac{1}{\tau }$. This damping rate comprises all energy losses of plasmon oscillations, (1) $\frac{1}{\tau_{0}}$—the losses caused by the electron scattering in the metallic nanoparticle on phonons, metal-crystal imperfections, other electrons and on the nanoparticle boundary, (2) $\frac{1}{\tau_{L}}$—the Lorentz friction losses corresponding to far-field zone irradiation (mostly in directions in the upper hemisphere for a nanoparticle deposited on a semiconductor substrate), (3) $\frac{1}{\tau^{\prime }}$—the energy losses corresponding to the energy transfer from plasmons to band electrons in the substrate semiconductor. Thus, $\frac{1}{\tau }=\frac{1}{\tau_{0}}+\frac{1}{\tau_{L}}+\frac{1}{\tau^{\prime }}$. We will show that the latter channel is the most effective one and practically all energy of surface plasmon is quickly, on the shortest time scale $\tau^{\prime }$, transfered to the semiconductor electrons. The Lorentz friction is also important, but only for larger metallic nanoparticles, a>15 nm (*a* is the nanosphere radius) [23,24]. The energy transfer, especially strong via near-field coupling of plasmons to band electrons in the semiconductor substrate, causes a strong damping of plasmons which stays behind the experimentally observed giant plasmon enhancement of the photo-effect [1,7,8,9,10,11,12,13,14,15,25,26]. According to the fluctuation–dissipation theorem, the strong irradiation (as of Lorentz friction type for large nanoparticles) is associated by also strong absorption rate of these nanoparticles—therefore they act as effective collectors for the incident sun-light even if their surface density in solar cell covering is low. Absorption and emission are in fact the same process with reversed time [19].

The surface plasmon oscillations have not-defined momentum, as localized oscillations. The band electrons are assigned, however, with momentum (quasi-momentum in crystal), the initial one, $p_{1}$, and the final one, $p_{2}$. They can be arbitrarily chosen because the momentum conservation is ruled out by the matrix element of the local dipole interaction for the plasmon-induced transition of electrons, contrary to the direct coupling with planar wave photons in the ordinary photo-effect (with condition $p_{1}=p_{2}$, strongly limiting the efficiency).

In the present paper, we summarize the theoretical description of the plasmon-aided photo-effect and compare the mostly analytical model with the numerical simulation of the metallically nano-improved solar cells upon the commercial numerical system Comsol. In the following paragraph, we provide the description of the Lorentz friction and the plasmon damping caused by these irradiation losses. In the next paragraph, we describe the channel of the energy transfer from plasmons to band-electrons in metallically improved solar cells, which is crucial for the efficiency enhancement of the plasmon photo-effect. Next, we provide the analysis of the nanoparticle size dependence of the plasmon photo-voltaic effect and draw out the conclusion for corrections for the Comsol applied to the plasmon photo-voltaic system simulation via inclusion of the appropriately lifted dielectric function of the metallic nanoparticles. The latter makes the Comsol simulation more realistic and suitable for comparison with the experiment, which we illustrate at the end.

The discussion presented in this paper found applications to photo-diodes covered with metallic nanoparticles or real solar cells metallically improved for which it is experimentally observed that there is a significant plasmon-induced increase of efficiency [7,8,13,27,28,29,30,31,32,33,34,35]. Typical metal materials are Au and Ag with surface plasmon resonances in nanoparticles overlapping with the visible light spectrum. Typical sizes of nanoparticles vary between 10 nm and 100 nm in diameter and the surface density of the metallic coverages is usually ca. $10^{8-10}$/cm${}^{2}$. It is surprising that such rarely dispersed small particles on the surface of a photo-cell can significantly enhance its photo-efficiency (e.g., twice in the photo-diode setup reported in [8]). This phenomenon corresponds to the exceptionally high radiative abilities of metallic nanoparticles of such size. Conveniently for metallic (Au, Ag, Cu) nanoparticles, the maximum of the surface plasmon damping due to irradiation (i.e., due to the Lorentz friction [17,18]) occurs at ca 50 nm for a radius of the nanosphere, which results in high irradiation and absorption rates of that size nanoparticles [24]. Thus, such nanoparticles very efficiently capture the incident light despite their low concentration. A very high concentration is inconvenient because of the reflection effect and destructive interference.

The possibility to improve efficiency of solar cells via some non-expensive and technologically feasible methods is now of large significance because of still not enough high efficiency of commercial solar cells. To increase this efficiency, various strategies are considered including, for example, quantum dot admixtures in multi-layer cells to better adjust absorption spectrum to the dispersion of the solar light on the earth surface or the metallic coverings of the photo-active surface of cells to mediate sun-light absorption via surface plasmons in metallic nanoparticles deposited on the substrate semiconductor. The plasmon-mediated photo-effect is the subject of our present analysis. We demonstrate that this effect has a quantum character and in order to meet the theoretical modeling with the experimental observations one must abandon the conventional classical methods of plasmonics which are offered by the solution of the Maxwell–Fresnel problem by utilization of e.g., the finite element method for solution of differential equations upon the commercial system Comsol, because the discrepancy of the latter simulation with the exact quantum approach (and with the experimental data) reaches several dozen percent. So a large error is caused by the negligence in classical methods of the very effective quantum channel for energy transfer between plasmons in metallic nanoparticles and band electrons in a semiconductor substrate. This channel can be accounted for in terms of the Fermi golden rule applied to inter-band transition of electrons in the semiconductor substrate induced by coupling to plasmons excited by the incident solar-light photons in metallic nanoparticles deposited on the photo-active surface. This channel for the energy transfer accurs to be highly effective and overwhelming the solar-light absorption in solar cells metallically modified in the nano-scale. The details of this strong plasmon photo-voltaic effect are presented in subsequent paragraphs.

## 2. Lorentz Friction Channel for Energy Losses of Surface Plasmons in a Metallic Nanoparticle

Plasmon oscillations in a metallic nanoparticles are widely analyzed by application of various methods, by the numerical Kohn–Sham-type approach [36,37] but ranged to ultra-small clusters only (because of the numerical calculus constraints), by random phase approximation approach (RPA) [15,22,38,39] and by the classical solution of the Fresnel–Maxwell equations [21]. The latter approach is usable for arbitrary size particles, but is limited by the negligence of quantum effects important in the nanoscale of metallic particles. The Mie approach [21], similarly as the numerical solution of the Fresnel–Maxwell boundary problem by the finite-element-method utilized by the Comsol system, suffers from the phenomenological only assumption of the dielectric function of metallic components as the prerequisite for the calculation algorithm. This dielectric function (in Drude form [21]) should comprise all quantum effects related to plasmons, but is not so because the assumed frequency and damping of plasmons is taken from bulk metal (at the best from measurements of thin films), but not of metallic nanoparticles. It has been demonstrated that the size effect for metallic nanoparticles is predominant for the range of nanoparticle radius a∈(15,100) nm (Au in vacuum) [23,24] resulting in a different Mie response and Comsol results in comparison to these models with the bulk dielectric function taken as the prerequisite for the calculus.

The reason of such astonishing observation is a consequence of the strongly growing irradiation losses of plasmons (i.e., the Lorentz friction losses) in the case of nanoparticles with a∈(15,100) nm (Au in vacuum, but similarly also for Ag and Cu and other dielectric surroundings), not present neither in smaller nanoparticles nor in the bulk metal.

**Figure 1:**
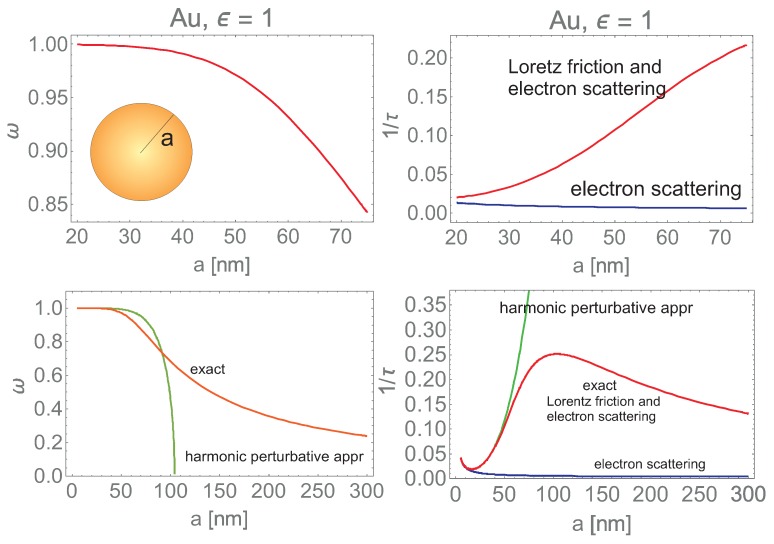
Size dependence of the resonance surface plasmon oscillations (dipole mode) including the Lorentz friction in the metallic (Au) nanoparticle in vacuum for size range, the radius a∈(20,70) nm (upper) and a∈(5,300) nm (lower). The inharmonic oscillation regime (caused by the third order time derivative for the Lorentz friction force [17,18])—red line—is apparently (cf. Equation (1)) strongly different than the harmonic approximation for the Lorentz friction—green line [23,24]. The overdamped regime does not occur in the exact inharmonic oscillations (red curve), whereas the approximate harmonic oscillations stop (green curve) when damping acquires 1/τ=1 (the resonance frequency, ω, and the damping rate, 1/τ, are expressed in dimensionless units, i.e., they are divided by $\omega_{1}$). In blue line it is extracted the electron scattering damping, which occurs much lower than the Lorentz friction losses for a>20 nm and diminishes with *a* growth.

To illustrate it, let us write out the resonance frequency and related damping obtained by the solution of the RPA dynamic equation for surface plasmons in metallic nanoparticle [15,23,24] with inclusion of the Lorentz friction force being proportional to the third-order time-derivative of the plasmon dipole [17,18]. The inharmonic behavior of the plasmon oscillations caused by the third-order time derivative in the Lorentz friction term is remarkable. The frequency and damping are no longer linked by the ordinary relation, $\omega =\omega_{1}\sqrt{1-{\left(\frac{1}{\tau_{0}\omega_{1}}\right)}^{2}}$, conventionally resulting in the overdamped regime without oscillations when $\frac{1}{\tau_{0}\omega_{1}}>1$. The exact solution including the Lorentz friction is always of the oscillatory type with the complex frequency,

(1) $$\begin{array}{cc} \omega +i\frac{1}{\tau }= & -\frac{i}{3l}+\frac{i\left(1+i\sqrt{3}\right)\left(1+6lq\right)}{3\times 2^{2/3}l{\left(2+27l^{2}+18lq+\sqrt{4{\left(-1-6lq\right)}^{3}+{\left(2+27l^{2}+18lq\right)}^{2}}\right)}^{1/3}}\\ & +\frac{i\left(1-i\sqrt{3}\right){\left(2+27l^{2}+18lq+\sqrt{4{\left(-1-6lq\right)}^{3}+{\left(2+27l^{2}+18lq\right)}^{2}}\right)}^{1/3}}{6\times 2^{1/3}l}, \end{array}$$

where $q=\frac{1}{\tau_{0}\omega_{1}}$ and $l=2/3{\left(\frac{a\omega_{1}}{c}\right)}^{3}$. The functions ω and $\frac{1}{\tau }$ (in dimensionless units, i.e., divided by $\omega_{1}$—the non-shifted by damping bare frequency) are plotted in Figure 1 versus the nanosphere radius *a*. Strong deviation from the harmonic behavior is apparent for a>40 nm. In Figure 1 the absence of the overadamped regime is sharply noticeable. The electron scattering losses, $\frac{1}{\tau_{0}}=\frac{v_{F}}{2l_{b}}+\frac{Cv_{F}}{2a}$ (where $v_{F}$ is the Fermi velocity in the metal, $l_{b}$ is the mean free path in the metal, C≃1 is the factor depending of the reflection type of electron scattering on the nanoparticle boundary) [15,40] are extracted in Figure 1, and is noticeable that this channel of losses is negligible for a>25 nm (Au in vacuum) in comparison to the Lorentz friction losses. It must be emphasized that in the conventional Mie and Comsol approaches [21] only $\frac{1}{\tau_{0}}$ is accounted for and this is a source of above mentioned discrepancies with the experimental observations.

It is clear that so strong plasmon damping as that one via the Lorentz friction channel must be included for a realistic description of plasmon phenomena in the scale of metallic nanoparticles, a∈(15,100) nm. If such nanoparticles are deposited on the semiconductor surface to mediate the photo-effect, the situation changes again in a pronounced manner. The coupling of plasmons with the substrate band electrons opens an especially efficient channel for the energy transfer and this channel dominates the plasmon damping—it is stronger than the above-described Lorentz friction damping. To describe this channel the quantum approach must be applied in the framework of the Fermi golden rule.

## 3. Fermi-Golden-Rule for Probability of Electron Inter-Band Excitation Due to Plasmons in Metallic Nanoparticle Deposited on a Semiconductor

**Figure 2:**
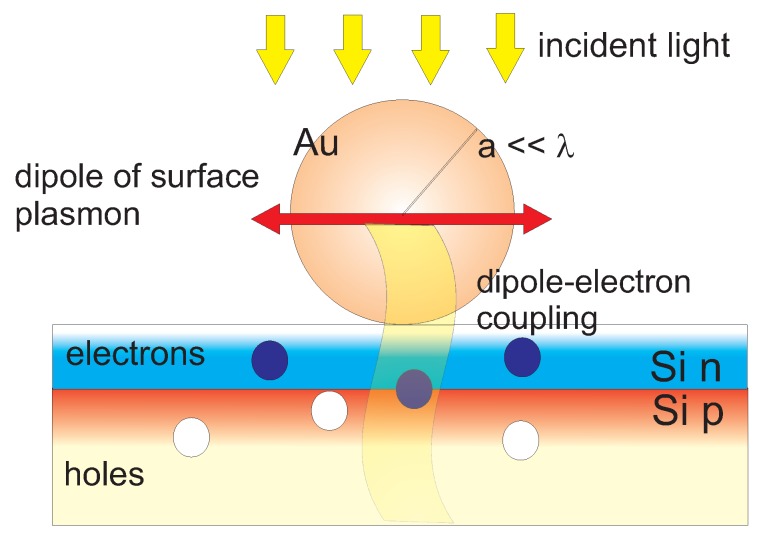
The spherical metallic nanoparticle (Au, Ag or Cu) with radius *a* is deposited on the semiconductor substrate (for example Si) with an active n-p junction embedded close (in depth <1μm) to the upper surface with the nanoparticle deposited. High plasmon-absorption rate of the nanoparticle with size a∈(10,70) nm (Au) [24] guaranties the high level excitation of the dipole plasmon mode by the incident light. The very effective channel for the energy transfer from the dipole-surface-plasmon to the band electrons is opened via near-field (and thus sub-photon, i.e., on the distance much lower than λ–the plasmon resonance wave length) coupling of dipole plasmon oscillations with band electrons in the semiconductor substrate, especially in the n-p junction, which results in a photo-current in a photo-diode configuration.

Let us consider now a metallic nanoparticle (of noble metal Au, Ag or of Cu) of spherical shape with radius a∈(5,70) nm deposited on the semiconductor substrate with embedded n-p junction for a setup of a photo-diode–as schematically presented in Figure 2.

We assume that in the metallic nanoparticle, it excites the surface plasmon of the dipole type. Such a plasmon is excited by incident photons (sun-light) with frequency close to the dipole-surface-plasmon resonance, which in Au (Ag or Cu) nanoparticles with radius of several tens of nanometers falls at resonance wave-length ∼500 nm, i.e., is highly greater than the nanoparticle dimension. Thus, the electrical component of the plasmon resonance e-m wave is almost homogeneous along the whole nanoparticle and the dipole regime is fulfilled, i.e., only the dipole mode of surface plasmons can be excited by this e-m wave.

Fourier components of the electric, $E_{\omega }$, and magnetic, $B_{\omega }$, fields produced in the distance R>a from the center of a nanosphere with the radius *a* and with the oscillating dipole of the surface plasmon, $D\left(t\right)∼D_{0}e^{-i\omega t}$, have the form [17,18],

(2) $$E_{\omega }=\frac{1}{\varepsilon }\left\{D_{0}\left(\frac{k^{2}}{R}+\frac{ik}{R^{2}}-\frac{1}{R^{3}}\right)+\hat{n}\left(\hat{n}\cdot D_{0}\right)\left(-\frac{k^{2}}{R}-\frac{3ik}{R^{2}}+\frac{3}{R^{3}}\right)\right\}e^{ikR}$$

and

(3) $$B_{\omega }=\frac{ik}{\sqrt{\varepsilon }}\left[D_{0}\times \hat{n}\right]\left(\frac{ik}{R}-\frac{1}{R^{2}}\right)e^{ikR},$$

where ε is the dielectric permittivity of the surroundings. In the case of the spherical symmetry of the metallic nanoparticle, the dipole of surface plasmon is considered as pinned to the center of the nanosphere (the origin of the reference frame system), though the dipole field is defined for R>a in the fully retarded form. In Equations (2) and (3) the notation for the retarded argument, $i\omega \left(t-\frac{R}{c}\right)=i\omega t-ikR$, $\hat{n}=\frac{R}{R}$, ω=ck, the momentum p=ℏk, is used. The terms with the denominators $R^{3}$, $R^{2}$ and *R* refer conventionally to the near-, medium- and far-field zones of the dipole radiation-field, respectively. It must be emphasized that in the near-field zone, when only terms with the denominator $R^{3}$ contribute (as the greatest at a<R<λ) the e-m wave is not yet formed (the magnetic field is zero). The e-m wave may be addressed to R>λ especially in the far-field zone where the locally planar wave picture is consistent with the ordinary k photon propagation. Thus, the near-field limit may be referred to as the sub-photonic region.

In the case of the metallic nanoparticle deposited on a semiconductor surface, the dominating interaction concerns a closely adjacent layer of the substrate semiconductor, thus terms with denominators $R^{2}$ and *R* may be neglected as small in comparison to the term with $R^{3}$ denominator. In the near-field zone the magnetic field disappears and the electric field is of the form of a static dipole field [17,18]. The related perturbation potential added to Hamiltonian of the band electron system in the substrate semiconductor attains in this case the form,

(4) $$\begin{array}{c} w=e\psi \left(R,t\right)=\frac{e}{\varepsilon R^{2}}\hat{n}\cdot D_{0}sin\left(\omega t+\alpha \right)=w^{+}e^{i\omega t}+w^{-}e^{-i\omega t}. \end{array}$$

The term $w^{+}={\left(w^{-}\right)}^{*}=\frac{e}{\varepsilon R^{2}}\frac{e^{i\alpha }}{2i}\hat{n}\cdot D_{0}$ corresponds to the emission and it is the case of our interest.

According to the FGR [19], the inter-band transition probability is proportional to

(5) $$w\left(k_{1},k_{2}\right)=\frac{2\pi }{\hbar }{\left|<k_{1}\left|w^{+}\right|k_{2}>\right|}^{2}\delta \left(E_{p}\left(k_{1}\right)-E_{n}\left(k_{2}\right)+\hbar \omega \right).$$

The Bloch states in the conduction and valence bands we assume here as planar waves for the simplicity reason (i.e., neglecting the Bloch periodic modulation function [16]), $\Psi_{k}=\frac{1}{{\left(2\pi \right)}^{3/2}}e^{ik\cdot R-iE_{n(p)}\left(k\right)t/\hbar }$, $E_{p}\left(k\right)=-\frac{\hbar^{2}k^{2}}{2m_{p}^{*}}-E_{g},E_{n}\left(k\right)=\frac{\hbar^{2}k^{2}}{2m_{n}^{*}}$, the indices n,p refer to electrons from the conduction and valence bands, respectively, $E_{g}$ is the forbidden gap, $m_{n}^{*}\left(p\right)$ defines the effective mass of electrons in the conduction (valence) band.

Conveniently, the matrix element

(6) $$<k_{1}\left|w^{+}\right|k_{2}>=\frac{1}{{\left(2\pi \right)}^{3}}\int d^{3}R\frac{e}{\varepsilon 2i}e^{i\alpha }\hat{n}\cdot D_{0}\frac{1}{R^{2}}e^{-i(k_{1}-k_{2})\cdot R}$$

can be analytically integrated, which results in the formula,

(7) $$\begin{array}{c} <k_{1}\left|w^{+}\right|k_{2}>=\frac{-1}{{\left(2\pi \right)}^{3}}\frac{ee^{i\alpha }}{\varepsilon }D_{0}cos\Theta \left(2\pi \right)\int_{a}^{\infty }dR\frac{1}{q}\frac{d}{dR}\frac{sinqR}{qR}\\ =\frac{1}{{\left(2\pi \right)}^{2}}\frac{ee^{i\alpha }}{\varepsilon }\frac{D_{0}\cdot q}{q^{2}}\frac{sinqa}{qa}, \end{array}$$

where $q=k_{1}-k_{2}$. The next step is the summation over all initial and all final states in both bands. Thus, for the total interband transition probability we obtain

(8) $$\delta w=\int d^{3}k_{1}\int d^{3}k_{2}\left[f_{1}\left(1-f_{2}\right)w\left(k_{1},k_{2}\right)-f_{2}\left(1-f_{1}\right)w\left(k_{2},k_{1}\right)\right],$$

where $f_{1},f_{2}$ assign the temperature dependent distribution functions (Fermi-Dirac distribution functions) for initial and final states, respectively. For room temperatures $f_{2}≃0$ and $f_{1}≃1$, which leads to

(9) $$\delta w=\int d^{3}k_{1}\int d^{3}k_{2}\cdot w\left(k_{1},k_{2}\right).$$

After the direct integration also in an analytical manner in the above formula, we arrive at the expression

(10) $$\begin{array}{c} \delta w=\frac{4}{3}\frac{\mu^{2}\left(m_{n}^{*}+m_{p}^{*}\right)2\left(\hbar \omega -E_{g}\right)e^{2}D_{0}^{2}}{\sqrt{m_{n}^{*}m_{p}^{*}}2\pi \hbar^{5}\varepsilon^{2}}\int_{0}^{1}dx\frac{sin^{2}\left(xa\xi \right)}{{\left(xa\xi \right)}^{2}}\sqrt{1-x^{2}}\\ =\frac{4}{3}\frac{\mu^{2}}{\sqrt{m_{n}^{*}m_{p}^{*}}}\frac{e^{2}D_{0}^{2}}{2\pi \hbar^{3}\varepsilon^{2}}\xi^{2}\int_{0}^{1}dx\frac{sin^{2}\left(xa\xi \right)}{{\left(xa\xi \right)}^{2}}\sqrt{1-x^{2}}, \end{array}$$

$\mu =\frac{m_{n}^{*}m_{p}^{*}}{m_{n}^{*}+m_{p}^{*}}$ is the reduced mass and the parameter is defined as $\xi =\frac{\sqrt{2\left(\hbar \omega -E_{g}\right)\left(m_{n}^{*}+m_{p}^{*}\right)}}{\hbar }$. In limiting cases for a nanoparticle radius *a*, we finally obtain

(11) $$\begin{array}{c} \delta w=\left\{\begin{array}{c} \frac{4}{3}\frac{\mu \sqrt{m_{n}^{*}m_{p}^{*}}\left(\hbar \omega -E_{g}\right)e^{2}D_{0}^{2}}{\hbar^{5}\varepsilon^{2}},\;\mathrm{for}\;a\xi \ll 1,\\ \frac{4}{3}\frac{\mu^{3/2}\sqrt{2}\sqrt{\hbar \omega -E_{g}}e^{2}D_{0}^{2}}{a\hbar^{4}\varepsilon^{2}},\;\mathrm{for}\;a\xi \gg 1. \end{array}\right. \end{array}$$

In the latter case in Equation (11) the following approximation has been applied:

$$\int_{0}^{1}dx\frac{sin^{2}\left(xa\xi \right)}{{\left(xa\xi \right)}^{2}}\sqrt{1-x^{2}}\approx \left(\mathrm{for}\;\;a\xi \gg 1\right)\frac{1}{a\xi }\int_{0}^{\infty }d\left(xa\xi \right)\frac{sin^{2}\left(xa\xi \right)}{{\left(xa\xi \right)}^{2}}=\frac{\pi }{2a\xi },$$

whereas in the former one $\int_{0}^{1}dx\sqrt{1-x^{2}}=\pi /4$.

With regard to two limiting cases, aξ≪1 or aξ≫1, $\xi =\frac{\sqrt{2\left(\hbar \omega -E_{g}\right)\left(m_{n}^{*}+m_{p}^{*}\right)}}{\hbar }$, we see that $a≃1/\xi ≃\left\{\begin{array}{c} >2\times 10^{-9}\left[m\right]\;for\;\frac{\hbar \omega -E_{g}}{E_{g}}<0.02\\ <2\times 10^{-9}\left[m\right]\;for\;\frac{\hbar \omega -E_{g}}{E_{g}}>0.02 \end{array}\right.$, and this range weakly depends on effective masses and $E_{g}$. Thus, for nanoparticles with radii a>2 nm the first regime holds only close to $E_{g}$ (less than the 2% of the distance of the ω to the limiting $E_{g}$), whereas the second regime holds in the rest of the ω domain. For the comparison, $a≃1/\xi ≃\left\{\begin{array}{c} >0.5\times 10^{-9}\left[m\right]\;for\;\frac{\hbar \omega -E_{g}}{E_{g}}<0.5\\ <0.5\times 10^{-9}\left[m\right]\;for\;\frac{\hbar \omega -E_{g}}{E_{g}}>0.5 \end{array}\right.$, the first region widens considerably (to ca. 50% of the relative distance to $E_{g}$), but holds only for ultra-small size of nanoparticles (a<0.5 nm). For larger nanospheres, e.g., with a>10 nm, the second regime is thus dominating.

One can notice that the above formula, Equation (10) and its explicit form in limiting situations given by Equation (11), is the generalization of to the ordinary photo-effect, for which the transition probability is different [16]:

(12) $$\begin{array}{c} \delta w_{0}=\frac{4\sqrt{2}}{3}\frac{\mu^{5/2}e^{2}}{m_{p}^{*2}\omega \varepsilon \hbar^{3}}\left(\frac{\varepsilon E_{0}^{2}V}{8\pi \hbar \omega }\right){\left(\hbar \omega -E_{g}\right)}^{3/2}. \end{array}$$

The number of photons of the ω e-m wave with the electric field component amplitude $E_{0}$ contained in the volume *V* equals to $\left(\frac{\varepsilon E_{0}^{2}V}{8\pi \hbar \omega }\right)$, hence, the probability of the single photon absorption by the semiconductor per time unit, attains the form in the ordinary photo-effect [16]:

(13) $$q_{0}=\delta w_{0}{\left(\frac{\varepsilon E_{0}^{2}V}{8\pi \hbar \omega }\right)}^{-1}=\frac{4\left(4\right)\sqrt{2}}{3}\frac{\mu^{5/2}e^{2}}{m_{p}^{*2}\omega \varepsilon \hbar^{3}}{\left(\hbar \omega -E_{g}\right)}^{3/2},$$

(factor (4) corresponds here to the spin degeneracy of band electrons).

In the case of the mediation by plasmons, all oblique interband transitions contribute, not only vertical ones (as it was for the interaction with the planar wave in the ordinary photo-effect). This results in the enhancement of the transition probability for the near-field coupling of plasmons with band-electrons in comparison to the photon (planar wave) absorption rate in a semiconductor in the ordinary photo-effect. The enhancement of the probability of the transition due to the admission of interband hopping not conserving momentum is, however, gradually quenched with the radius *a* growth, as expressed by Equation (11).

The probability of energy absorption in the semiconductor via mediation of surface plasmons counted per single photon incident on the metallic nano-spheres, $q_{m}$, equals to the product of δw (given by Equation (11)) and the number, $N_{m}$, of metallic nanoparticles divided by photon density with additional phenomenological factor β (called as the proximity factor) responsible for all effects not directly accounted for (as a deposition separation and surface properties reducing the coupling strength),

(14) $$q_{m}=\beta N_{m}\delta w{\left(\frac{\varepsilon E_{0}^{2}V}{8\pi \hbar \omega }\right)}^{-1}.$$

## 4. Damping Rate for Plasmons in a Metallic Nanoparticle Deposited on the Top of a Semiconductor

Assuming that the energy acquired by the semiconductor band system, A, is equal to the output of the plasmon oscillation energy (resulting in plasmon damping), one can estimate the corresponding damping rate of plasmon oscillations. Namely, at the damped (lowering in time) plasmon amplitude $D_{0}\left(t\right)=D_{0}e^{-t/\tau^{\prime }}$, one finds for a total transmitted energy:

(15) $$A=\beta \int_{0}^{\infty }\delta w\hbar \omega dt=\beta \hbar \omega \delta w\tau^{\prime }/2=\left\{\begin{array}{c} \frac{2}{3}\frac{\beta \omega \tau^{\prime }\mu \sqrt{m_{n}^{*}m_{p}^{*}}\left(\hbar \omega -E_{g}\right)e^{2}D_{0}^{2}}{\hbar^{4}\varepsilon^{2}},\;\mathrm{for}\;a\xi \ll 1,\\ \frac{2}{3}\frac{\beta \omega \tau^{\prime }\mu^{3/2}\sqrt{2}\sqrt{\hbar \omega -E_{g}}e^{2}D_{0}^{2}}{a\hbar^{3}\varepsilon^{2}},\;\mathrm{for}\;a\xi \gg 1, \end{array}\right.$$

where $\tau^{\prime }$ is the damping time-rate, β accounts for losses not included in the model. Comparing the value of A given by the Formula (15) with the energy loss of damping plasmon estimated in [15] (the initial energy of the plasmon oscillations which has been transferred step-by-step to the semiconductor, $A=\frac{D_{0}^{2}}{2\varepsilon a^{3}}$), one finds

(16) $$\frac{1}{\tau^{\prime }}=\left\{\begin{array}{c} \frac{4\beta \omega \mu \sqrt{m_{n}^{*}m_{p}^{*}}\left(\hbar \omega -E_{g}\right)e^{2}a^{3}}{3\hbar^{4}\varepsilon },\;\mathrm{for}\;a\xi \ll 1,\\ \frac{4\beta \omega \mu^{3/2}\sqrt{2}\sqrt{\hbar \omega -E_{g}}e^{2}a^{2}}{3\hbar^{3}\varepsilon },\;\mathrm{for}\;a\xi \gg 1. \end{array}\right.$$

By $\tau^{\prime }$ we denote here a short time-scale for the large damping, $1/\tau^{\prime }$, of plasmons due to the energy transfer to the semiconductor substrate highly exceeding the internal (in metal) damping, characterized by $1/\tau_{0}$, the latter due to the scattering of electrons inside the metallic nanoparticle [15] ($\frac{1}{\tau_{0}}\ll \frac{1}{\tau^{\prime }}$). The irradiation to the far-field zone toward the upper hemisphere (i.e., the Lorentz friction for the plasmon) is also smaller than the near-field zone energy transfer to the substrate [15].

## 5. The Efficiency of the Plasmon-Mediated Photo-Effect

To describe the plasmon-mediated photo-effect one must consider the scenario when the output of the plasmon energy is recovered by the continuous energy income from the incident sun-light. This leads to the energy-balance regime, when the sun-light energy flows through plasmons to the substrate semiconductor at stationary conditions (the ordinary photon absorption by the semiconductor must be also included, in a realistic situation). In an idealized case, the whole incoming energy of the monochromatic ω e-m wave is transferred to the semiconductor via plasmons, and we deal with the stationary state of a driven and damped oscillator for plasmons. Despite the free undamped plasmons have the Mie self-resonance frequency, $\omega_{1}=\frac{\omega_{p}}{\sqrt{3}}$, the frequency of driven and damped plasma oscillations equals to the driven electric field frequency, ω, of the incident e-m wave of photons. Because of an instant leakage of the plasmon energy in the near field to the semiconductor substrate, the resulted large damping of plasmon causes a red-shift and a widening of the resonance, as for every damped and driven oscillator. The widened resonance enables the energy transfer from plasmons to electrons to embrace also frequencies different than the bare Mie frequency, but limited from below by the semiconductor forbidden gap $E_{g}/\hbar$.

The incident sun-light dispersion covers the visible spectrum and also some UV and infra-red tails. The total efficiency of the plasmon channel corresponds to a sum (integration) over all Fourier components $\omega >E_{g}/\hbar$ of light with intensity tuned by the distribution in sun-light spectrum. To model this behavior it is necessary to consider separately each single monochromatic e-m mode, i.e., each Fourier component ω. The electric field of ω e-m wave excites plasmon with the same frequency ω and this plasmon is damping with the rate $\frac{1}{\tau^{\prime }}$ given by Equation (16). This damping causes a red-shift of the resonance and reduces the resonance amplitude, which in turn allows for the accommodation to the balance of the energy transfer to the semiconductor with the incident sun-light e-m wave energy intensity (defined by the e-m electric field amplitude $E_{0}$) at the frequency ω. Within this damped and driven oscillator model, the amplitude of plasmon oscillations $D_{0}\left(\omega \right)$ is constant in time and shaped by $f\left(\omega \right)=\frac{1}{\sqrt{{\left(\omega_{1}^{2}-\omega^{2}\right)}^{2}+4\omega^{2}/\tau^{{}^{\prime }2}}}$. The extreme of red-shifted resonance is attained at $\omega_{m}=\omega_{1}\sqrt{1-2{\left(\omega_{1}\tau^{\prime }\right)}^{-2}}$ with the corresponding amplitude $∼\tau^{\prime }/\left(2\sqrt{\omega_{1}^{2}-\tau^{{}^{\prime }-2}}\right)$. The red-shift is proportional to $1/(\omega_{1}\tau^{{}^{\prime }2})$. In the case of the described energy transfer balance one obtains according to Equation (11)

(17) $$q_{m}=\left\{\begin{array}{c} \beta C_{0}\frac{128}{9}\pi^{2}a^{3}\frac{\mu \sqrt{\mu_{n}^{*}\mu_{p}^{*}}}{m^{2}}\left(\hbar \omega -E_{g}\right)\frac{e^{6}n_{e}^{2}\omega }{\hbar^{4}\varepsilon^{3}}f^{2}\left(\omega \right),\;\mathrm{for}\;a\xi \ll 1,\\ \beta C_{0}\frac{128}{9}\sqrt{2}\pi^{2}a^{2}\frac{\mu^{3/2}}{m^{2}}\sqrt{\hbar \omega -E_{g}}\frac{e^{6}n_{e}^{2}\omega }{\hbar^{3}\varepsilon^{3}}f^{2}\left(\omega \right),\;\mathrm{for}\;a\xi \gg 1, \end{array}\right.$$

where $f\left(\omega \right)=\frac{1}{\sqrt{{\left(\omega_{1}^{2}-\omega^{2}\right)}^{2}+4\omega^{2}/\tau^{{}^{\prime }2}}}$ corresponds to the amplitude factor for the driven damped oscillator and $D_{0}=\frac{e^{2}n_{e}E_{0}4\pi a^{3}}{3m}f\left(\omega \right)$ (in Equation (11)); the amplitude of the electric field, $E_{0}$, in the incident e-m wave is next ruled out from Equation (17) due to the normalization per single photon as in Equation (14); $C_{0}=\frac{N_{m}4/3\pi a^{3}}{V}$, *V* is the volume of the semiconductor, $N_{m}$ is the number of metallic nanospheres.

The ratio, $\frac{q_{m}}{q_{0}}$, revealing the advantage of the plasmon-mediated photo-effect over the ordinary photo-effect can be expressed as follows:

(18) $$\frac{q_{m}}{q_{0}}=\left\{\begin{array}{c} \frac{4\sqrt{2}\pi^{2}a^{3}\beta C_{0}\sqrt{m_{n}^{*}m_{p}^{*}}{\left(m_{p}^{*}\right)}^{2}e^{4}n_{e}^{2}\omega^{2}f^{2}\left(\omega \right)}{3\mu^{3/2}m^{2}\sqrt{\hbar \omega -E_{g}}\hbar \varepsilon^{2}},\;\mathrm{for}\;a\xi \ll 1,\\ \frac{8\pi^{2}a^{2}\beta C_{0}{\left(m_{p}^{*}\right)}^{2}e^{4}n_{e}^{2}\omega^{2}f^{2}\left(\omega \right)}{3\mu m^{2}\left(\hbar \omega -E_{g}\right)\varepsilon^{2}},\;\mathrm{for}\;a\xi \gg 1. \end{array}\right.$$

This ratio turns out to be of order of $10^{4}\frac{\beta 40}{H[nm]}$ for the surface density of nano-particles (as in the experiment reported in [8]), $n_{s}∼10^{8}$/cm${}^{2}$; note that $C_{0}=n_{s}4\pi a^{3}/\left(3H\right)$ (in Equation (18)), *H* is the thickness of the semiconductor layer. Including the phenomenological factor β and the thickness *H* (we have confirmed experimentally that the range of the near-field zone exceeds the Mie wave-length, i.e., is not shorter than 1 μm) the above formula is sufficient to explain the scale of the experimentally observed strong enhancement of the absorption rate in semiconductors due to plasmons. The strong enhancement of this transition probability is linked with the admission of momentum-not-conserved transitions, which is, however, reduced with the radius *a* growth. The strengthening of the near-field induced inter-band transitions, in the case of large nano-spheres, is, however, still significant as the quenching of oblique interband transitions is partly compensated by ∼$a^{3}$ growth of the amplitude of dipole plasmon oscillations. The trade-off between these two competing size-dependent factors is responsible for the observed experimentally enhancement of light absorption and emission in diode systems mediated by surface plasmons in nano-particle surface coverings [7,8,9,26,41].

To illustrate the above described strengthening of the photo-effect by plasmon-mediation one can estimate the photo-current in the case of a semiconductor photodiode without and with the metallically modified photo-active surface. This photo-current is $I^{\prime }=\left|e\right|N\left(q_{0}+q_{m}\right)A$, where *N* is the number of incident photons and $q_{0}$ and $q_{m}$ are the probabilities of single photon absorption in the ordinary photo-effect [16] and of single photon absorption mediated by the presence of metallic nano-spheres, respectively, as derived above; $A=\frac{\tau_{f}^{n}}{t_{n}}+\frac{\tau_{f}^{p}}{t_{p}}$ is the amplification factor ($\tau_{f}^{n(p)}$ is the annihilation time of both sign carriers, $t_{n(p)}$ is the drive time for carriers [the time of traversing the distance between the electrodes]). From the above definitions, it follows that the efficiency measure for the plasmon photo-voltaic effect attains the form (here $I=I^{\prime }\left(q_{m}=0\right)$, i.e., the photo-current without metallic colorred modifications)

(19) $$\frac{I^{\prime }}{I}=1+\frac{q_{m}}{q_{0}},$$

where the ratio $q_{m}/q_{0}$ is given by Equation (18).

**Figure 3:**
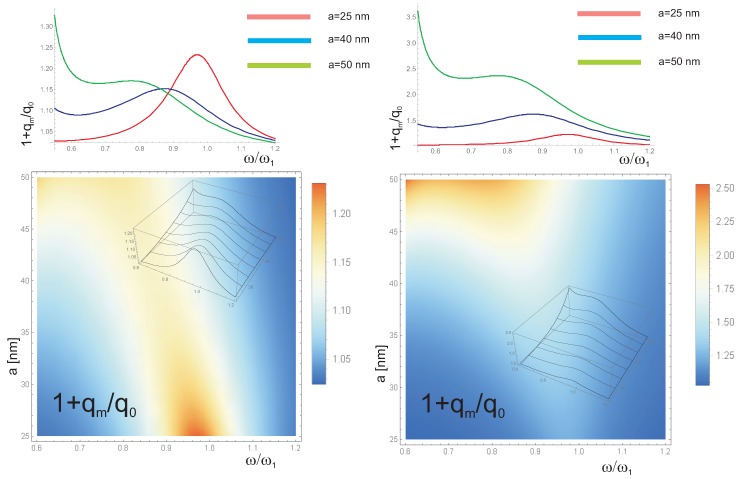
Size dependence of the photo-effect efficiency enhancement due to plasmon-mediation in light absorption, (**left**) for constant total mass of all metallic nanoparticles deposited on the semiconductor (Si) substrate and (**right**) for constant surface concentration of particles (the layer with the thickness H=3μm, the proximity factor β=0.0005, the density $n_{s}=50\times 10^{8}{\left(\frac{25}{a}\right)}^{3}$/cm${}^{2}$ (**left**) and $n_{s}=50\times 10^{8}$/cm${}^{2}$**(right**), of Au nanoparticles with radius a∈(25,50) nm). The red shift with the nanoparticle size growth is noticeable. The efficiency increase $I/I_{0}=1+q_{m}/q_{0}$ (cf. Equations (19) and (18)) depends on the metallic nanoparticle size, material metal and semiconductor parameters and on the density of the metallic coverage (ω is the frequency of the incident light in dimensional units, i.e., divided by $\omega_{1}$).

All material parameters as well as the geometry and size parameters modify both Equations (18) and (17) which gives the eventual relatively complicated dependence of the plasmon-mediated photo-effect efficiency $\frac{q_{m}}{q_{0}}\left(\omega \right)$ with respect to the material, size and the deposition type. We illustrate this dependence in Figure 3.

The Formula (18) is exemplified in Figure 3 for Au nanoparticles deposited on an Si semiconductor which reproduces well the experimental behavior [8] (the required material data are listed in Table 1 and Table 2). Both channels of photon absorption resulting in photo-current in the semiconductor sample are included, the direct ordinary photo-effect absorption with probability of transitions is given by $q_{0}$ and the plasmon-mediated absorption with probability $q_{m}$, respectively. Note also that some additional effects like the reflection of incident photons or the destructive interference on metallic net would contribute and it was phenomenologically accounted for in the plasmon-mediated channel by an experiment-fitted factor β. The collective interference type corrections are rather not strong for the considered low densities of metallic coverings of the order of $10^{9}$/cm${}^{2}$, and nano-sphere sizes well lower than the resonant wave-length (∼500 nm), though for larger concentrations and larger nano-sphere sizes, they would play a stronger reducing role (reflecting photons) [13,28]. The resonance threshold was accounted for by the damped resonance envelope function in Equation (19) including the semiconductor band-gap limit. As indicated in Figure 3, the relatively high value of $\frac{q_{m}}{q_{0}}∼10^{4}\frac{\beta 40}{H[nm]}$ enables a significant growth of the efficiency of the photo-energy transfer to a semiconductor mediated by surface plasmons in nano-particles deposited on an active layer, by increasing β or reducing *H* (at constant $n_{s}$). However, because of the fact that an enhancement of β easily induces the overdamped regime of plasmon oscillations; the more effective prospective would be lowering of *H* especially convenient in thin film solar cells. This reflects the fact that the damping of plasmons due to coupling with band electrons in the substrate semiconductor can highly exceed the Lorentz friction (the latter can be reduced by the fully embedding of the nanoparticle in a semiconductor). The electron scattering losses do not play any role in comparison to the dominant channel of the energy flow. The overall behavior of $I^{\prime }/I=1+q_{m}/q_{0}$ calculated according to the relation (19), and depicted in Figure 3, agrees well with the experimental observations [8], in the position, height and shape of the photo-current curves for distinct samples.

**Figure 4:**
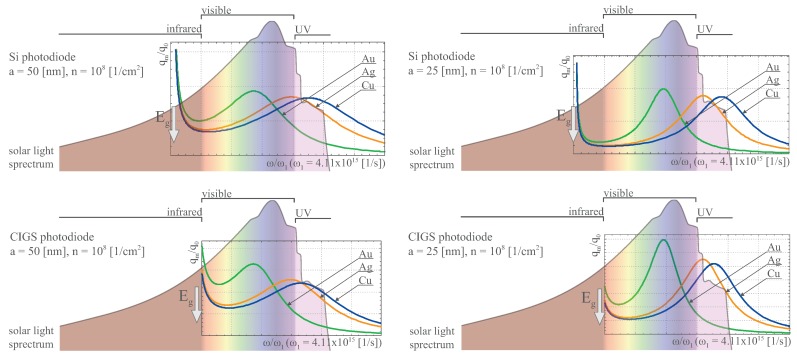
Comparison of the effectiveness of the plasmon channel expressed by $\frac{q_{m}}{q_{0}}\left(\omega \right)$ (cf. Equation (18)) for the same substrate semiconductor Si (**upper**) and CIGS (**lower**) with Au (red), Ag (blue) and Cu (green) nanoparticles of the same radius of 50 nm (**left**), 25 nm (**right**) and the same surface density $10^{8}$/cm${}^{2}$, versus the sun-light spectrum on the earth surface—the figure illustrates accommodation of the spectral characteristics of the plasmon-mediated photo-effect, $\frac{q_{m}}{q_{0}}\left(\omega \right)$, to sun-light spectrum for different materials and covering parameters; arrows indicate the positions of the forbidden gap for Si and CIGS, respectively, marked on the background of the solar light spectrum; the latter accommodated to the frequencies $\omega /\omega_{1}$ on the horizontal axis, the vertical axis shows the efficiency growth on the same scale for Au, Ag and Cu not related to the vertical height of the solar spectrum.

We have compared the spectral dependence of the plasmonic efficiency enhancement with respect to the substrate change (Si, CIGS—copper-indium-gallium-diselenide) for the same Au, Ag and Cu nanoparticles with radius a=50,25 nm and the same nanoparticle concentration $n_{s}=10^{8}$/cm${}^{2}$. From this comparison, in Figure 4, for Si and CIGS substrates with Au, Ag, Cu nanoparticles of size a=50,25 nm (at the nanoparticle concentration $n_{s}=10^{8}$/cm${}^{2}$) one can notice that Au nanoparticles utilize the visible sun-light spectrum in the better manner than Ag or Cu ones. The advantage of Au nanoparticles is greater in the case of Si substrate and is reduced for CIGS substrate because the blue-shift of $E_{g}$ in CIGS with respect to Si. In the case of CIGS (especially for larger nanoparticles, a=50 nm), the advantage of Au beyond Ag in overall utilization of sun-light spectrum disappears, whereas is pronounced in the case of Si substrate. This is because of the cut-off of the near infra-red part of sun-light in spectral absorption of CIGS in contrary to Si, and in favor to Ag more than to Au nanoparticles, cf. Figure 4. This behavior agrees with the experimental observation of Si and CIGS substrates covered by Au and Ag nanoparticles [28].

For nanoparticles of gold (Au) and silver (Ag) of size a=50 nm, optimized due to Formula (18), deposited on the multi-crystalline silicon (mc-Si) and on the copper-indium-gallium-diselenide (CIGS) solar cells, the measured [28] overall increase of cell efficiency attains the level of even 5%. The application of suitable concentration of Au and Ag nanoparticles onto mc-Si solar cells increases their efficiency by 5.6% and 4.8%, respectively [28]. Application of Au and Ag nanoparticles onto surfaces of CIGS solar cells improves their efficiency by 1.2% and 1.4%, respectively [28].

## 6. Numerical Modeling of Plasmon Photo-Effect by Comsol

The commercial numerical packet Comsol is a convenient tool for the solution by the finite-element method of differential equation systems with imposed arbitrary boundary conditions. It can be utilized to the solution of the Maxwell–Fresnel problem at the arbitrary geometry and material composition of analyzed systems (cf. Comsol Multiphysics 5.0, Wave Optics module, http://www.comsol.com). In particular, solar cells can be modeled in such a way, including plasmonic effect induced by the metallic nanoparticles deposited on a cell surface. To solve the Maxwell–Fresnel equation system for a cell, the appropriate geometry arrangement of the system is required including the predefinition of all material parameters, i.e., of the dielectric functions for all components of the investigated device.

The dielectric function defines the displacement electric field D(ω,k)=ε(ω,k)E(ω,k) and comprises information about all processes involved in the light matter interaction including microscopic quantum material properties, expressed, however, in an effective macroscopic manner in the real and imaginary parts of the dielectric function in the Fourier domain for space–time variables. This allows for inclusion of the microscopic effects within the completely classical calculus of Maxwell–Fresnel boundary problem.

The energy dissipation inside a dispersive material can be calculated according to the formula (in the uniform system case) [17],

(20) $$Q=\frac{\omega }{4\pi }\left(\varepsilon^{{}^{\prime \prime }}E^{2}+\mu^{{}^{\prime \prime }}H^{2}\right),$$

where the $\varepsilon^{{}^{\prime \prime }}$ and $\mu^{{}^{\prime \prime }}$ are the imaginary parts of the permittivity and permeability, respectively. For solar cells, the magnetic properties are unimportant, so the dielectric function $\varepsilon =\varepsilon^{{}^{\prime }}+\varepsilon^{{}^{\prime \prime }}$ is of the central importance. The real and the imaginary parts of ε are connected to the refractive index and the absorption coefficient, respectively. In particular,

(21) $$\varepsilon^{{}^{\prime \prime }}\left(\omega \right)=\frac{nc}{\omega }\alpha \left(\omega \right)$$

where *n* is the refractive index and α is the absorption coefficient. The absorption coefficient,

(22) α(ω)∼ℏωδω

where the photon absorption probability in the case of the ordinary photo-effect is given by Equation (12), i.e.,

(23) $$\delta \omega_{0}=\frac{4\sqrt{2}}{3}\frac{\mu^{5/2}e^{2}}{m_{p}^{2}\omega \varepsilon \hbar^{3}}\left(\frac{4\pi \varepsilon_{0}\varepsilon E_{0}^{2}V}{8\pi \hbar \omega }\right){\left(\hbar \omega -E_{g}\right)}^{3/2},$$

and in the case of the photo-effect mediated by metallic nanoparticles, by Equation (11),

(24) $$\delta \omega =\frac{4}{3}\frac{\mu^{3/2}\sqrt{2}\sqrt{\hbar \omega -E_{g}}e^{2}D_{0}^{2}}{a16\pi^{2}\varepsilon_{0}^{2}\varepsilon^{2}\hbar^{4}}.$$

For Comsol calculus convenience, the plasmon dipole amplitude $D_{0}$ can be evaluated according to the formula describing the total power irradiated by an oscillating dipole:

(25) $$D_{0}^{2}=\frac{4\pi \varepsilon_{0}\lambda^{4}}{{\left(2\pi \right)}^{4}c}P=\frac{4\pi \varepsilon_{0}\lambda^{4}}{{\left(2\pi \right)}^{4}c}\int_{\Sigma }S\cdot d\sigma ,$$

where *P* is the total power irradiated by the dipole, S is the Poynting vector and Σ is the nanoparticle surface.

The total energy of light absorbed to the semiconductor substrate can be calculated by the integration of the square of the electric field over the semiconductor volume,

(26) $$Q_{with(without)}\left(\omega \right)=\frac{\omega }{4\pi }\int_{V}n_{s}\varepsilon_{with(without)}^{{}^{\prime \prime }}\left(\omega \right)E^{2}dV,$$

where $\varepsilon^{{}^{\prime \prime }}$ is the imaginary part of the dielectric function of the Si substrate with and without metallic nanoparticles deposited, respectively (‘without’ will be equivalently noted as ‘0’). The light absorption enhancement (the efficiency enhancement) can be then defined, as the ratio of the light absorbed by the semiconductor covered with metallic nanoparticles and the one without any metallic coverage,

(27) $$A\left(\omega \right)=\frac{Q_{with}\left(\omega \right)}{Q_{0}\left(\omega \right)}.$$

The enhancement of the induced short circuit current $I_{sc}$ in the cell (due to the presence of metallic nanoparticles) can be calculated by the integration of the absorption enhancement A over the solar spectrum:

(28) $$I_{sc}=\frac{\int Q_{with}\left(\omega \right)F\left(\omega \right)d\omega }{\int Q_{0}\left(\omega \right)F\left(\omega \right)d\omega },$$

where F(ω) is the standard solar global spectrum, assumed as the conventional AM1.5 G.

**Figure 5:**
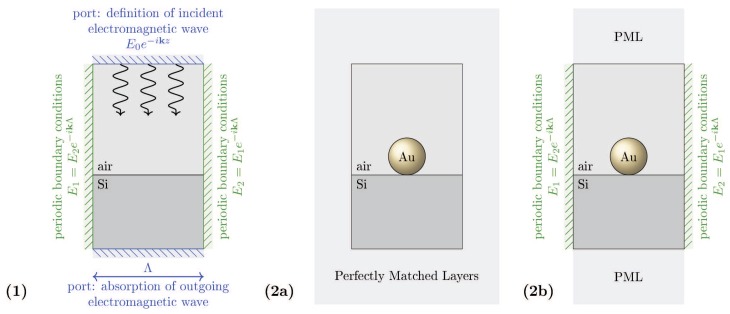
The scheme of the unit cell of numerical model for Comsol. (**1**) The first step of calculations—the evaluation of the background electric field as the reference field distribution for the second step. (**2a**) The second step for the single particle model (none periodic conditions are imposed). (**2b**) The second step for the model of metallic nanoparticles (MNPs) array with the periodic Floquet conditions imposed.

The scheme of the calculation model set-up (the unite sell for Comsol calculation) is shown in Figure 5. The simulation unit cell consists of three domains: (1) the air-surroundings, (2) the semiconductor substrate, and (3) the metallic nanoparticle. In order to investigate the wide range of possible concentrations of the metallic coverage (including the regimes of e-m non-coupled and e-m coupled metallic nanoparticles in the coverage array), we have considered two models: (1) the model of a single metallic nanoparticle on the substrate (which can be next multiplied by the nanoparticle concentration, neglecting, however, all inter-particle e-m interaction) and (2) the model of periodic metallic nanoparticle array deposited on the semiconductor substrate (allowing for the inclusion of the interparticle e-m interference being the function of the particle separation).

For both model definitions, the calculus is carried out in two steps. In the first step, we calculate the background electric field distribution, i.e., the electric field distribution in the case of the plane electromagnetic wave incoming onto the bare semiconductor surface. In the second step, this field distribution serves as the reference distribution for the evaluation of the scattered electric field arising due to the presence of the metallic nanoparticles deposited on the semiconductor surface.

The first step in both model arrangements is identical. We have set the incident light as a plane wave propagating vertically along the z-axis direction and polarized parallel to the semiconductor surface, by using two ports. At the top boundary, we define the incoming plane wave parameters and at the bottom boundary, the parameters for the wave transmitted through the substrate. On the lateral boundaries we have defined the Floquet periodic boundary conditions, which allowed us to effectively simulate a large system (in the case of the duplication of the unite cell according to the particle surface concentration which mimics the multiparticle coverage with e-m interaction between metallic components homogeneously distributed on the semiconductor substrate surface).

For the single particle model (for independent particle case), in the second step, we have defined additional domain surrounding the calculation cell and absorbing all outgoing light (so-called perfectly matched layers (PML)), instead of the periodic conditions. This allow us to reduce the simulation area, which is important for time minimization of the numerical algorithm. This model treats the nanoparticles as completely independent, which is relevant only to low concentrations of the coverage. The width of the computation cell was set to 350 nm, the hight of the air-surroundings is set to 300 nm, the Si substrate thickness is set to 200 nm and PML thickness to 150 nm.

For the model of the periodic array of metallic nanoparticles, in the second step, we use again the Floquet periodic boundary conditions on the lateral boundaries and set the additional PML at the top and at the bottom of the computational cell. The width of the computational cell is assumed to variate in the range Λ∈(90,360) nm, the hight of the air-surroundings is set to 300 nm, Si substrate thickness to 400 nm and PML thickness to 150 nm.

Outside of the PML domains, we use tetrahedral mesh elements with the size equal to $\frac{a}{5}$ inside the metallic nanoparticle of the radius *a*, $\frac{\lambda }{30}$ inside the Si substrate and $\frac{\lambda }{6}$ inside the air-surroundings domain (λ is the wave-length of the incident light).

We have performed calculations with the dielectric function for Si modification in the absorption including its part by the formula found by ourselves (in the framework of the Fermi Golden Rule, cf. Equation (11)) and have compared the results with the similar calculations using the experimentally taken dielectric function for Si without any metallic modification, taken from [42]. The dielectric function of the Au was modeled in the Drude approximation with the damping rate given by Equation (16).

**Figure 6:**
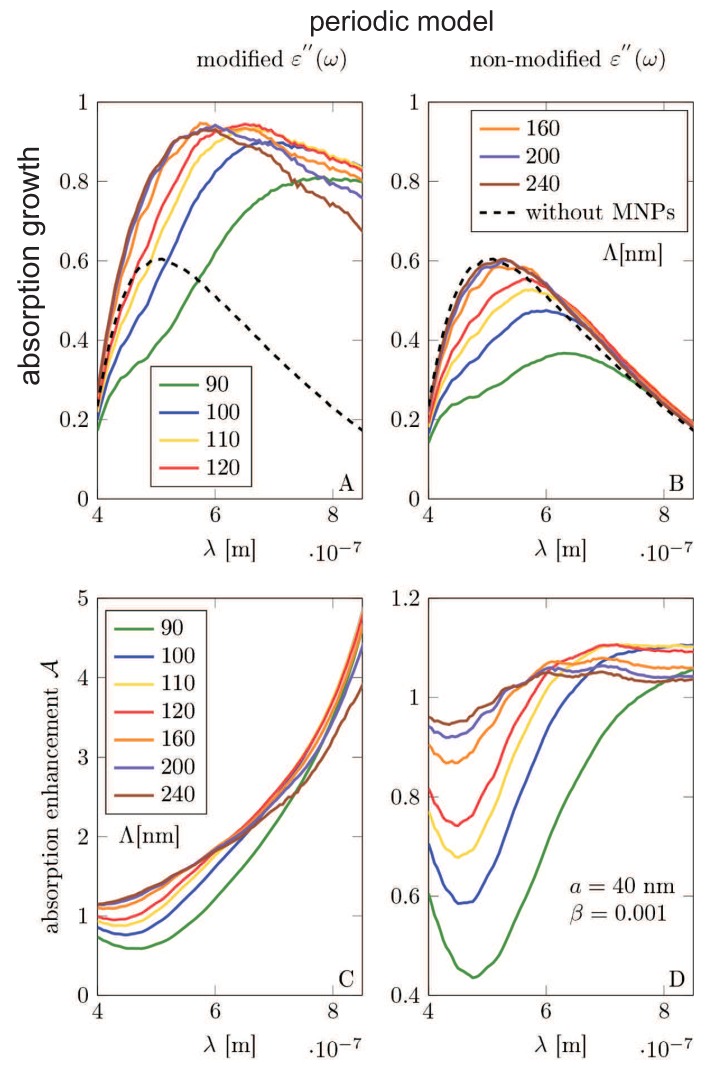
The periodic model—(**A**,**B**): The absorption enhancement of the light absorption in the Si substrate as the function of the wavelength of incident electromagnetic wave for various size of the unite cell Λ. (**C**,**D**): The efficiency enhancement of the system acc Equation (27) as a function of the wavelength of incident electromagnetic wave. (**A**,**C**): results obtained in the model using the modified dielectric function of the semiconductor $\varepsilon^{{}^{\prime \prime }}\left(\omega \right)$ by plasmon damping contribution (the energy leaving plasmons incomes to the semiconductor substrate); (**B**,**D**): results obtained in the model using non-modified dielectric function of the semiconductor measured in bulk [42]. The calculations was made for MNPs of radii a=40 nm and for the array period Λ=90,100,110,120,160,200,240 nm describing the lowering of the nanoparticle concentration.

**Figure 7:**
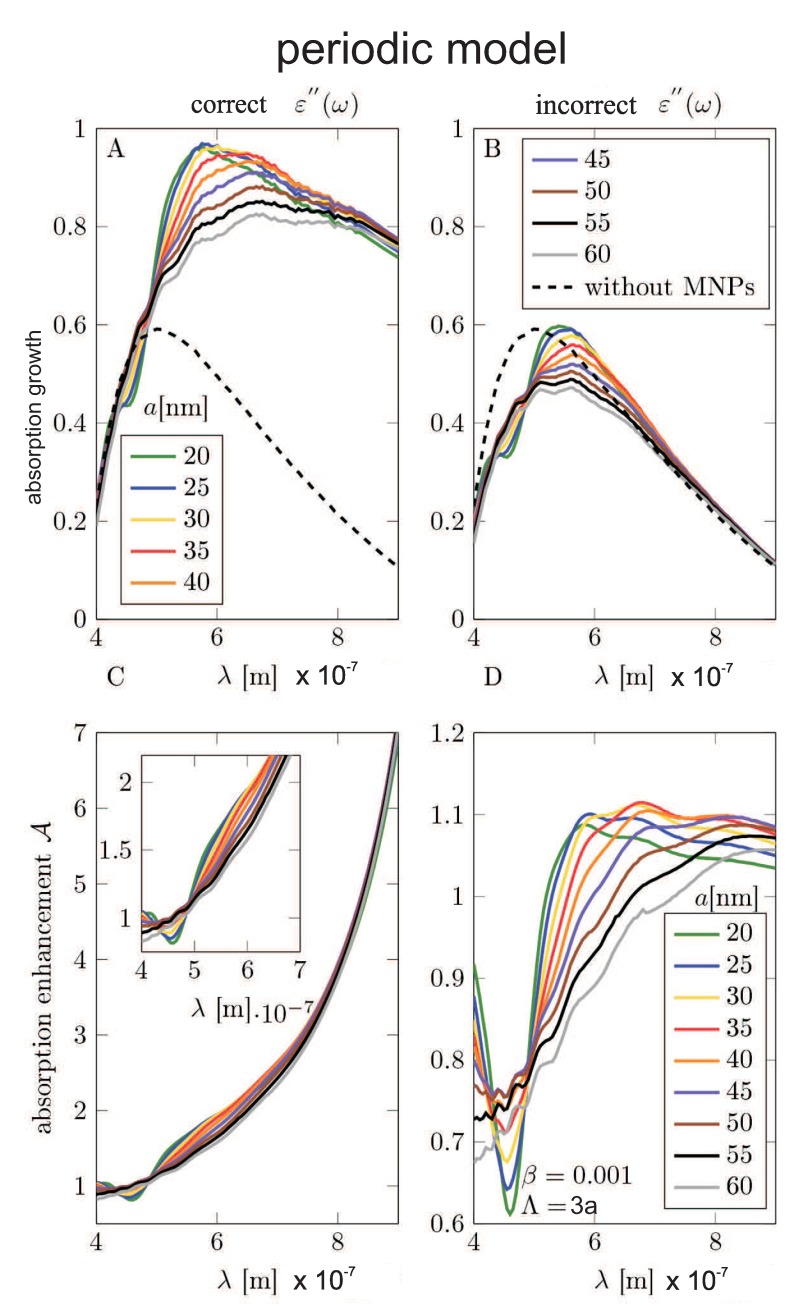
The periodic model—(**A**,**B**): The absorption enhancement in the Si substrate as the function of the wavelength of incident electromagnetic wave for varying radius of metallic nanoparticles. (**C**,**D**): The efficiency rate growth acc. Equation (27) as a function of the wavelength of incident electromagnetic wave for varying radius of nanoparticles. (**A**,**C**): The results obtained in the model using modified $\varepsilon^{{}^{\prime \prime }}\left(\omega \right)$; (**B**,**D**): The results obtained in the model using inappropriate (non-modified) dielectric function taken from the measurement in bulk [42]. The calculation was made for MNP arrays with the period equal Λ=3a and the radius a=20,25,30,35,40,45,50,55,60 nm.

**Figure 8:**
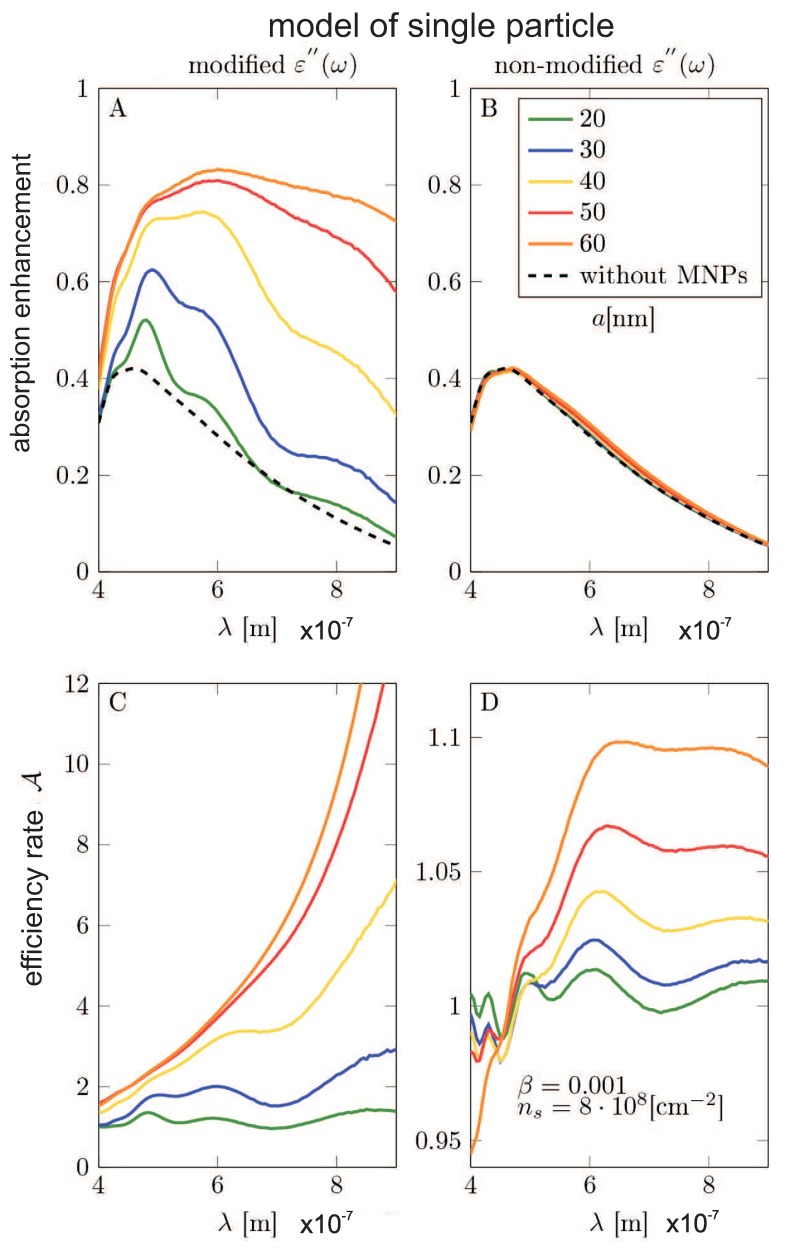
Single particle model—(**A**,**B**): The absorption growth in the Si substrate as a function of the wavelength of incident electromagnetic wave for varying radius of metallic nanoparticles (Λ accommodated to conserve surface density $n_{s}=8\times 10^{8}$/cm${}^{2}$). (**C**,**D**): The efficiency enhancement factor acc. Equation (27) as a function of the wavelength of incident electromagnetic wave for varying size of nanoparticles and constant their concentration. (**A**,**C**): Results obtained in the model using modified $\varepsilon^{{}^{\prime \prime }}\left(\omega \right)$. (**B**,**D**): Results obtained in the model using non-modified irrelevant dielectric function measured in bulk [42]. The calculations were made for on Si substrate deposited single MNP with radii a=20,30,40,50,60 nm, β=0.001 and for MNP effective (accommodated by Λ) concentration $n_{s}=8\times 10^{8}$/cm${}^{2}$.

**Figure 9:**
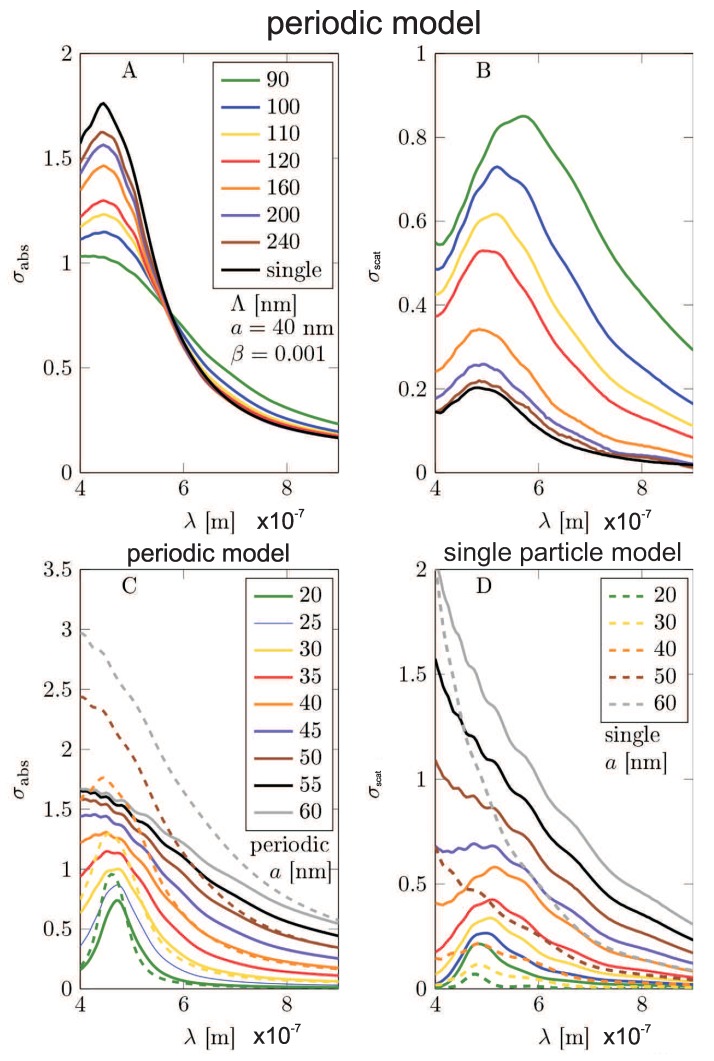
The periodic model—(**A**,**C**): The absorption cross section of Au NP deposited on Si substrate as a function of the wavelength of incident electromagnetic wave for varying single cell size (**A**) and varying nanoparticle radius (**B**). (**B**,**D**): The scattering cross section for Au NP deposited on Si substrate; the corresponding cross sections obtained in the case of the single particle model indicated by dashed lines. (**A**,**B**): Results obtained from the model of periodic MNP array with constant MNP radii a=40 nm and array period taken from the set Λ=90,100,110,120,160,200,240 nm.; (**C**,**D**): Results obtained from the model of periodic MNP array with varying MNPs radii a=20,25,30,35,40,45,50,55,60 nm and the array period Λ=3a. All the cross sections are normalized to the particle surface area.

**Figure 10:**
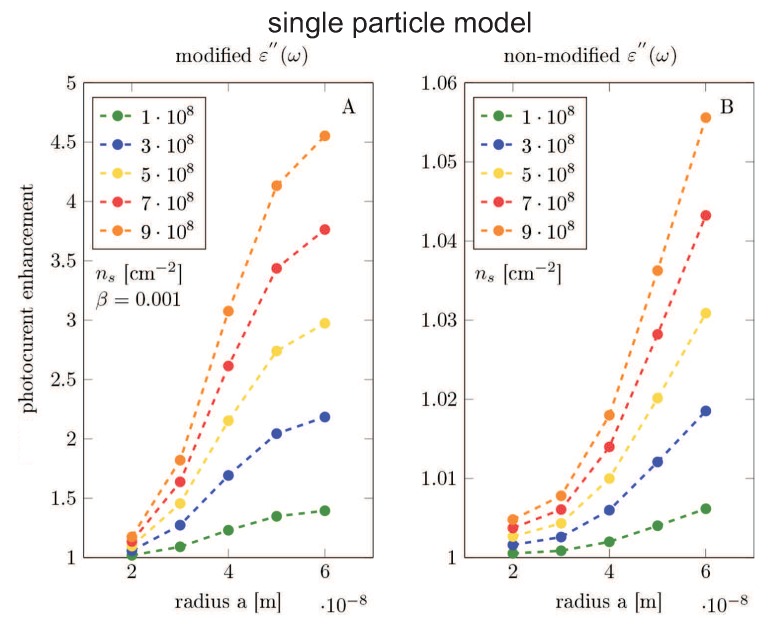
The single particle model—(**A**,**B**): The photocurrent enhancement (Equation (28)) as a function of MNP radius calculated for the model of the single MNP on Si substrate. The photocurrent enhancement is showed for various MNPs concentrations $n_{s}$. We used solar spectra AM1.5G. (**A**): Results obtained in the model using the modified $\varepsilon^{{}^{\prime \prime }}\left(\omega \right)$ by the plasmon damping. (**B**): Results obtained in the model using non-modified inappropriate dielectric function taken from the measurement in bulk [42].

**Figure 11:**
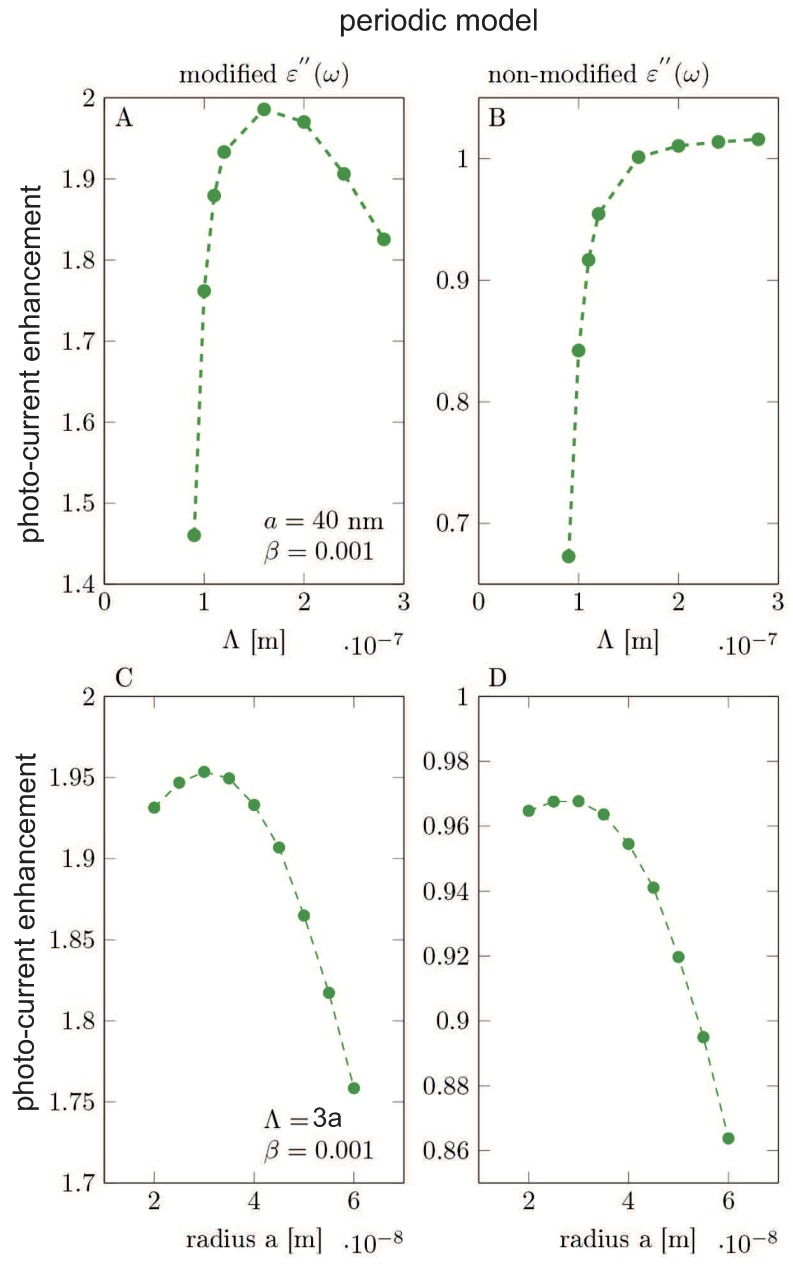
The periodic model (**A**,**B**): The photocurrent enhancement acc. to Equation (28) as a function of MNP array period. The radius of the MNP is assumed as a=40 nm. (**C**,**D**): The photocurrent enhancement as a function of MNPs radius. (**A**,**C**): Results obtained in the model using modified $\varepsilon^{{}^{\prime \prime }}\left(\omega \right)$. (**B**,**D**): Results obtained in the model using non-modified dielectric function taken from the measurement in bulk [42]. We used the solar spectrum AM1.5G.

The results of the Comsol calculation are illustrated in Figure 5, Figure 6, Figure 7, Figure 8, Figure 9, Figure 10 and Figure 11 and in Table 3 and Table 4. It is evidently noticeable that the negligence in simulation of the plasmon damping causes the reducing of the efficiency of the photo-effect by at least one order of magnitude, both in the model of the single particle (i.e., neglecting e-m interaction between particles)—cf. Figure 8 and Figure 10 and in the model of the periodic array (including near-neighbor e-m interaction)—Figure 6, Figure 7, Figure 9, Figure 11. The effect is similarly strong in the value for the absorption in the Si substrate including mediation of nanoparticles (Figure 6, Figure 7 and Figure 8) with respect to the nanoparticle radius or the calculation of cell size (changing of which is equivalent to the variation of the nanoparticle concentration) and in the value of the absorption cross section refined for nanoparticles (also versus size parameters, Figure 11). For the photo-current, the strengthening achieves even two orders of magnitude in comparison to only electro-magnetic effects of the field concentration close to nanoparticles according to the Maxwell–Fresnel solution without quantum corrections (cf. Figure 10 for the single particle model). Some explicit quantitative comparison is exemplified in Table 3 for the calculus, including quantum corrections versus a similar calculus without quantum corrections—Table 4. This comparison agrees with the more comprehensive presentation in Figure 6, Figure 7, Figure 8, Figure 9, Figure 10 and Figure 11. It is noticeable also that there is an agreement with analytical analysis, acc. to Equations (19) and (18) illustrated in Figure 3. The similar red shift of the plasmonic photo-effect strengthening is visible in the Comsol simulations—cf. Figure 7, as well as the same concerns the monotonic dependence of the efficiency enhancement rate with respect to the nanoparticle concentration. Some advantage of the Comsol simulation is, however, noticeable in its ability to account for the e-m interaction of nanoparticles (confined, however, to nearest neighbors only via simple periodic conditions imposed in the model of the periodic array of nanoparticles). The reducing role of the strong density of the metallic coverage (probably due to collective reflection) is noticeable in a simulation of the periodic array (Figure 6, Figure 7, Figure 9, Figure 11). The observation that this reducing role is strongly weakened by the inclusion of quantum corrections related to the described above giant plasmon damping is interesting.

We have confined the presented simulation and former analytical theory to spherical-shaped metallic nanoparticles. For Comsol, there is, however, no difference to simulate an arbitrary shape nanoparticle, including an arbitrary type deposition on the semiconductor substrate (in particular, including the case of a nanoparticle partly embedded in a semiconductor medium). Such flexibility of the Comsol calculus is its great advantage. We expect that the variation of the nanoparticle shape would not cause significant changes with respect to the behavior described for spherical nanoparticles, because the shape modification influences only the self-modes of dipole surface plasmons, which slightly shifts the resonance frequency, practically without any perturbation of the plasmon damping due to coupling with band electrons. The latter can be strongly changed, however, by the type of the nanoparticle deposition. For example, the near-field coupling of plasmon dipole mode with band electrons in the semiconductor can be heavily enhanced if the nanoparticle would be completely embedded in a semiconductor medium instead of only being deposited on the semiconductor surface. Practical realization of such a setup may be arranged by the sandwich structure with metallic nanoparticles located in-between semiconductor layers. Though such an experiment has not been performed, one can expect further growth of the plasmon-mediated photo-effect due to the strengthening of coupling of plasmon to electrons in the surrounding semiconductor from all directions.

## 7. Comparison with Experiment

A strong enhancement of the photo-effect induced by mediation of light energy transfer to the semiconductor substrate by surface plasmons in metallic nanoparticles deposited on the photo-active surface has been observed in various setups. Some of these observations are collected in Table 5. In practice, various methods of nanoparticle covering manufacturing are applied. For instance, in Refs. [43,44] surface supported gold nanoparticles are produced by pulsed laser deposition. The size of particles is controlled by the number of ablation shots and the gas pressure. Another method is by magnetron sputtering production of Au:TiO${}_{2}$ films which follows post-deposition annealing [45]. The incorporation of Ag:Zn nano-particles in the perovskite based solar cell [46,47] led to improved device performance attributed to the occurrence of local surface plasmon resonance of metal nano-composite resulted in solar cell efficiency enhancement from 4.52% to 5.70%. Hence, the incorporation of bi-metallic nano-particles in perovskite-based solar cells is a promising strategy for improving both stability and power conversion efficiency. Cs-doping is also considered to improve perovskite solar cells [48]. Large increases of perovskite cell efficiency have been reported (ca. 30%) after application of the Ag nano-components [49]. Nano-modifications of dye-solar-cells are also investigated [50], by carefully tuning the amount of organic fluorophore in the hybrid coating material, a maximum increase in power conversion efficiency exceeding 4% is achieved in flexible organic cell incorporating the new coating layer [51]. Various metals for nano-components have been also tested including e.g., aluminium and titanium besides gold and silver [52,53], as well as multi-shape particles (Ag) [54].

## 8. Conclusions

We have demonstrated that the negligence of quantum corrections in the nano-scale conventional numerical modeling of solar cells improved with metallic nanoparticles via the solution of Maxwell–Fresnel differential equations with a boundary condition (as e.g., employing the commercial packet Comsol) causes the giant inaccuracy which dismisses such modeling unless the quantum corrections are included. The quantum corrections concern the damping rate of plasmons mediating photo-effect in the case of the solar cell covered with relatively rarely dispersed metallic nanoparticles on their surface. In the case of the conventional modeling of such systems by application of the numerical algorithms (for the solution of Maxwell–Fresnel problem via the finite-element method), the dielectric functions of all materials in the complex setup are predefined as the prerequisite for the calculus. These material characteristics are taken usually from the experiment to model the dielectric functions for the semiconductor and the metal, the latter being in terms of the Drude approximation. The experimental measurements of the dielectric function are typically carried out in bulk and independently for each of the components. However, the microscopic coupling of the components in the complex system, especially the coupling of plasmons in the metallic nanoparticles with the band electrons in the substrate semiconductor (in the case of the metallized solar cells), changes significantly the material dielectric functions for both mutually coupled components in comparison to their individual characteristics when they were separated.

By application of the quantum Fermi Golden Rule scheme we have demonstrated that the efficiency of the energy transfer channel between the surface plasmon oscillations in metallic nanoparticles and a substrate semiconductor is big and sharply depends on parameters of both components (the radius and material and surface concentration of metallic nanoparticles and the energy gap, effective masses of electrons and holes and the permittivity of a semiconductor substrate). The related ultra-short-time energy transfer causes the large damping of plasmons and the remarkable increase of the efficiency of metallized solar cells observed in many experiments.

We have found the analytical formula for the plasmon-photo-effect efficiency and the time-scale of the related plasmon damping, which generalizes the ordinary photo-effect onto the plasmon-mediated one and agrees well with the experimental measurements in a laboratory photo-diode configuration. The measured ratio of photo-currents in the setup with and without metallic nano-components is compared with the theoretically predicted scenario. The quantitative consistence is achieved both for the shape of the spectral characteristics and for the particle-size dependence with the experiments with Si photodiode covered with Au nanoparticles with radii of few tens of nm and surface density $10^{8-9}$/cm${}^{2}$. The qualitative agreement has been also demonstrated for complete solar cells (multicrystal-Si and CIGS [copper-indium-gallium-diselenide]) where the plasmon effect is obscured by other elements of the long series of factors resulting in the overall solar cell efficiency besides the efficiency of the absorption of photons only. The increase of the overall photovoltaic efficiency for considered by us metallically modified cells varies between 1.5% (CIGS) and 6% (Si), depending on the nanoparticle concentration (for too dense concentrations the efficiency drops down, due to a destructive interference and reflection).

We have shown that the mediation of plasmons in metallic nanoparticles deposited on the surface of the photodiode can enhance the efficiency of the photo-effect even by a factor of 2 (i.e., of 100% increase), which has been confirmed experimentally. Such a large increase is, however, reduced in commercial Si solar cells to ca. 5% of overall efficiency increase (and to ca. 2% in CIGS cells). By the numerical modeling of the plasmon-mediated photo-effect upon the Comsol system, we have proved that the results strongly differ when the damping of plasmons is or is not included into the predefined dielectric functions of mutually coupled metallic and semiconductor components of the whole system. By the series of modelings upon various conditions, we have demonstrated that the negligence of the plasmon damping rate makes it difficult to fit the experimental data, whereas the inclusion of the plasmon damping rate allows for reasonable fitting. The difference is large; the efficiency enhancement found by Comsol without quantum corrections (i.e., when only concentration of the electrical field close to the curvature of metallic nanoparticles is accounted for by solution of the classical Maxwell–Fresnel problem) does not exceed 0.1, whereas the inclusion of the proper plasmon damping rises this factor at least by a one order of the magnitude.

Thus, we can conclude that the conventional numerical modeling (as upon the Comsol system) is strongly erroneous (at least by one order of magnitude for the relative efficiency increase) if carried out with the negligence of quantum-induced modifications of the dielectric functions of components of metallically nano-modified solar cells. We have shown, via comparison of the conventional Comsol simulation utilizing its commercial packets with the improved simulation along the described quantum scheme, that the conventional Comsol simulation fails in front of the experimental observations of the plasmon-mediated photo-effect. The realistic explanation of this effect is possible exclusively upon the developed quantum approach because the discrepancy between the conventional Comsol simulation of the efficiency of the plasmonic photo-effect and the related experimental data reaches at least one order of magnitude. This discrepancy is vanished by inclusion of the quantum corrections along the presented approach.

## Figures and Tables

**Table 1:** Mie frequency $\omega_{1}$ to Formula (18).

Metal	Au	Ag	Cu
$\omega_{1}$	$4.11\times 10^{15}$ 1/s	$5.2\times 10^{15}$ 1/s	$5.7\times 10^{15}$ 1/s

**Table 2:** Substrate material parameters to Formula (18) ($m=9.1\times 10^{-31}$ kg, the mass of bare electron; lh–light holes, hh–heavy holes, L–longitudinal, T–transverse).

Semiconductor	$m_{n}^{*}$	$m_{p}^{*}$	$E_{g}$
Si	0.9m L[101], 0.19m T[110]	0.16m lh, 0.49m hh	1.12 eV
GaAs	0.067m	0.08m lh, 0.45m hh	1.35 eV
CIGS	0.09–0.13 *m*	0.72m	1–1.7 eV

**Table 3:** The photocurrent enhancement for model of MNP array on Si substrate (acc. Equation (28)), for radii of MNPs a=20,30,40,50,60 nm and the array periods Λ=3a,4a. Results obtained in the model using the correctly modified dielectric functions in their imaginary part. We used solar spectra AM1.5G to calculate the photocurrent enhancement acc. to Equation (28).

Radius [nm]	Λ=3a	Λ=4a
20	1.66	1.44
30	1.87	1.71
40	1.97	1.91
50	1.98	1.97

**Table 4:** The photocurrent enhancement for the model of MNP array on Si substrate. The radius of MNPs was taken, a=20,30,40,50,60 nm and the array periods, Λ=3a,4a. The listed results are obtained in the model using the semiconductor dielectric function measured in bulk [42] and the dielectric function of metal without the proper plasmon damping. The solar spectrum AM1.5G.

Radius [nm]	Λ=3a	Λ=4a
20	0.996	0.999
30	1.006	1.010
40	1.011	1.014
50	1.006	1.014

**Table 5:** Measured values of the photo-current enhancement in silicon solar cells and silicon photo-diodes with deposited metallic nanoparticles. For various setups and nanoparticles deposition parameters the different increase of the photo-effect efficiency has been observed experimentally as reported in the indicated references. Majority of the observed behavior cannot be explained by only local concentration of the e-m field near the curvature of nanoparticles accounted for by the conventional Comsol modeling and the consistence of the experimental data with the theoretical simulation needs inclusion of the plasmon damping contribution as described in the present paper. However, some exceptionally low efficiency increase (or even decrease) evidences the complicated competition of various factors beyond the model considered in the paper, being apparently sensitive to the position of the p-n junction active layer in the substrate or to the interference destructive effects or reflection from too dense coverings.

Type	Size [nm]	Shape	Concentration	Enhancement	p-n Junction Depth [nm]	Device Type	Ref.
Au	50	spherical	$6.6\times 10^{8}$ [cm${}^{-2}$]	18% (max ${}^{1}$:80%)			
Au	80	spherical	$1.6\times 10^{8}$ [cm${}^{-2}$]	31% (max ${}^{1}$:100%)	80	c-Si	[8]
Au	100	spherical	$0.77\times 10^{8}$ [cm${}^{-2}$]	38% (max ${}^{1}$:60%)			
Au	100	spherical	$9.9\times 10^{8}$ [cm${}^{-2}$]	2.8%	500	c-Si ${}^{2}$	[27]
Au	100	spherical	$0.3\times 10^{8}$ [cm${}^{-2}$]	5.6%			
Au	100	spherical	$1.5\times 10^{8}$ [cm${}^{-2}$]	2.0%			
Au	100	spherical	$3.2\times 10^{8}$ [cm${}^{-2}$]	3.3%	>500 ${}^{3}$	mc-Si ${}^{4}$	[28]
Au	100	spherical	$6.67\times 10^{8}$ [cm${}^{-2}$]	−2.8%			
Au	100	spherical	$10\times 10^{8}$ [cm${}^{-2}$]	−7.5%			
Au	100	spherical	$3.5\times 10^{8}$ [cm${}^{-2}$]	3.3%	500	c-Si ${}^{5}$	[29]
Au	diameter 20, height [2;3]	island	$1.3\times 10^{11}$ [cm${}^{-2}$]	20% (max ${}^{1}$: 40%)	-	a-Si:H/c-S ${}^{6}$	[13]
Au	diameter 65	spherical	$10\cdot 10^{8}$ [cm${}^{-2}$]	18%	300	c-Si ${}^{7}$	[30]
Ag	40	island	$124\times 10^{8}$ [cm${}^{-2}$]	127%	160	SOI ${}^{8}$	
Ag	66	island	$67\times 10^{8}$ [cm${}^{-2}$]	283%	160	SO ${}^{8}$	[31]
Ag	108	island	$25\times 10^{8}$ [cm${}^{-2}$]	592%	160	SOI ${}^{8}$	
Ag	thickness 12, 14, 16 ${}^{9}$	island	-	19%, 14%, 2% ${}^{10}$	-	Si	[32]
Ag	diameter [120;140], height [45;60]	island	$30\times 10^{8}[$cm${}^{-2}$]	354%	95	SOI ${}^{8}$	[7]
Ag	thickness 12 and 16 ${}^{9}$	island	-	33% and 16% ${}^{10}$	1250	SOI ${}^{11}$	[32]
Ag	25:91	spheroidal	38.29%	17%		c-Si ${}^{12}$	
Al	22:81	spheroidal	40.40%	21%	-	c-Si ${}^{12}$	[35]
In	17:25	spheroidal	30.78%	23%	-	c-Si ${}^{12}$	
Ag	∼60	island	31%	0%	>150 ${}^{13}$	c-Si	[33]
Al	diameter 190, height 70	cylindrical	19.6% ${}^{14}$	49%		c-Si ${}^{15}$	[34]

${}^{1}$ The maximal value of absorption enhancement per single wavelength; ${}^{2}$ Commercialy available n/p Si solar cell produced by Silicon Solar, Inc.; ${}^{3}$ Emiter thickness is 500 nm; ${}^{4}$ Anti-reflection coating from SiN of thickness 70 nm; ${}^{5}$ N-type Si wafer (001) of thickness 300 mm and donor concentration ca. 1015 cm${}^{-3}$; ${}^{6}$ Heterojunction a-Si:H/c-Si (p-type, (100)). The thickness of a-Si:H layer is 18 nm; ${}^{7}$ Si wafer with transparent graphen electrode; ${}^{8}$ Silicon-on-insulator (SOI); ${}^{9}$ Nanoparticle coverage was fabricated by annealing of silver film of a particular thickness (12, 14, 16 nm) in the temperature 200 °C by 50 min; ${}^{10}$ Enhancement averaged over the solar spectrum AM1.5G; ${}^{11}$ Silicon-on-insulator (SOI): top Si layer thickness 1250 nm; ${}^{12}$ Solar cell covered by 20 nm layer of TiO${}_{2}$ separating nanoparticles form silicon; ${}^{13}$ Emiter thickness is 150 nm; ${}^{14}$ periodic, period 380 nm; ${}^{15}$ Solar cell covered by 40 nm layer of SiO${}_{2}$ separating nanoparticles from silicon.
